# Mce1C and Mce1D facilitate *N. farcinica* invasion of host cells and suppress immune responses by inhibiting innate signaling pathways

**DOI:** 10.1038/s41598-020-71860-8

**Published:** 2020-09-10

**Authors:** Xingzhao Ji, Xiujuan Zhang, Lina Sun, Xuexin Hou, Jingdong Song, Xiaoluo Tan, Han Song, Xiaotong Qiu, Minghui Li, Lu Tang, Lichao Han, Zhenjun Li

**Affiliations:** 1 Shandong Academy of Clinical Medicine, Provincial Hospital Affiliated to Shandong First Medical University, Jinan, 250000 China; 2 State Key Laboratory for Infectious Disease Prevention and Control, National Institute for Communicable Disease Control and Prevention, Chinese Center for Disease Control and Prevention, 155 Changbai Road Changping District, Beijing, 102206 China; 3 Department of Endocrinology, Beijing Chaoyang Hospital, Capital Medical University, Beijing, China; 4 Chenzhou Center for Disease Control and Prevention, Chenzhou, China; 5 National Institute for Viral Disease Control and Prevention, Chinese Center for Disease Control and Prevention, Beijing, China; 6 First Affiliated Hospital of Chengdu Medical College, Chengdu, China

**Keywords:** Tumour-necrosis factors, Bacterial pathogenesis

## Abstract

The mammalian cell entry (Mce) family of proteins consists of invasin-like membrane-associated proteins. The roles of Mce1C and Mce1D proteins in host–pathogen interactions have not been investigated. In this study, we demonstrate that Mce1C and Mce1D protein is localized in the cell wall fraction of *N. farcinica*. Both *N. farcinica* Mce1C and Mce1D proteins are expressed at the level of protein and mRNA and elicit antibody responses during infection. Mce1C and Mce1D facilitate the internalization of *Escherichia coli* expressing Mce1C protein or latex beads coated with Mce1D protein by HeLa cells, respectively. We further demonstrate that Mce1C and Mce1D can suppress the secretion of the proinflammatory factors TNF-α and IL-6 in macrophages infected with *Mycobacterium smegmatis* expressing Mce1C or Mce1D and promote the survival of *M. smegmatis* expressing Mce1C or Mce1D in macrophages. In addition, Mce1C and Mce1D supress the activation of the NF-κB and MAPK signaling pathways by blocking the phosphorylation of AKT, P65, ERK1/2, JNK, or P38 in macrophages. These findings suggest that Mce1C and Mce1D proteins facilitate *N. farcinica* invasion of HeLa cells and suppress host innate immune responses by manipulating NF-κB and MAPK signaling pathways, which may provide a target for *N. farcinica* treatment.

## Introduction

*Nocardia* is an intracellular gram-positive pathogenic bacteria that is partially acid-fast, aerobic, catalase positive, and ubiquitous in the environment^1^. There are more than 80 recognized *Nocardia* species, with 33 being responsible for human diseases^2,3^. Nocardiosis is mainly an opportunistic infection, and it affects immunocompromised hosts in up to 60% of cases, causing severe, life-threatening disseminated infections^4,5^.

Bacterial adhesion and invasion are considered important virulence factors in the infection processes^6^. Several experiments showed that *Nocardia* could cause the damage of cell and tissue by adhering and invading into host cells^7,8^ and the *Nocardia* within macrophages can even prevent the fusion of phagosomes–lysosomes, inhibit proteasome activity, resist oxidative killing, and survive in macrophages persistently^9,10^. Subsequent studies suggested that Mce, which is considered an essential virulence component of *Micobacterium tuberculosis*, may play a critical role in the process of *M. tuberculosis* invasion^11,12^. Ishikawa et al.^13^ showed that the *N. farcinica* genome contains six dispersed *mce* operons (*mce*1–6) that are homologous to the *M. tuberculosis* mce operon^14^. Mce1A, Mce1E, Mce3A, Mce3C, Mce3D, and Mce3E from *M. tuberculosis* are expressed and elicit antibody responses in naturally infected patients^15–18^. Previous studies have demonstrated that the virulence of *Nocardia brasiliensis* strains in BALB/c mice was completely lost after virulence factors were lost, including a group of Mce proteins^19^. Our earlier reports showed that Mce1E may facilitate *N. farcinica* interactions with and invasion of mammalian cells and could be expressed by *N. farcinica* during infection^18^.

Innate immunity constitutes the first line of defense against pathogen infection^20^. NF-κB and MAPK signaling pathways can regulate innate immune responses by controlling the expression of various inflammatory cytokines, such as TNF and IL-6, etc^21–23^ and pathogens could suppress those signaling pathways to subvert the early innate immune response^24^. P38 kinase, extracellular-regulated kinase (ERK)1/2, and c-Jun-N-terminal kinase (JNK) are the three types of MAPKs that can be activated independently or simultaneously^25^. *M. tuberculosis* Mce2E and Mce3E have been demonstrated to regulate innate immune response by inhibiting the MAPK signaling pathways and suppressing the expression of proinflammatory cytokines, such as TNF-α and IL-6^20,26^.

However, there is no study on the function of *N. farcinica* Mce1C and Mce1D proteins. Whether the Mce1C and Mce1D proteins in *N. farcinica* have the ability to facilitate *N. farcinica* invasion of host cells and are immunogenic remains unknown. In addition, relatively little is known about the role of the mce1 operon in interactions between *N. farcinica* and macrophages, especially the function of regulating host innate immune responses. In this study, we demonstrate that Mce1C and Mce1D proteins could promote *N. farcinica* invasion of mammalian cells. We also show that Mce1C and Mce1D were expressed during *N. farcinica* infection and could elicit antibody response. Further, we demonstrate that Mce1C and Mce1D suppress proinflammatory cytokine expression and promote the survival of *M. smegmatis* expressing Mce1C or Mce1D in macrophages. Also, Mce1C and Mce1D can regulate innate immunity by inhibiting activation of the NF-κB and MAPK signaling pathways. This is the first time that a related study was carried out on *N. farcinica* Mce1C and Mce1D proteins. Our findings indicate that Mce1C and Mce1D are *N. farcinica* virulence factors and thus may contribute to the study of the interaction between *Nocardia* and host.

## Materials and methods

### Bacterial strains, cells, and culture conditions

Five clinical isolates of *N. farcinica* and the standard strain *N. farcinica* IFM10152 were included in the present study. The five strains were collected from different hospitals (strains of CDC33, CDC43, CDC142, CDC146, and CDC154 collected from Gan Su, Bei Jing, Hai Nan, Guang Xi, and Jiang Su, respectively), and the standard strain was purchased from the German Resource Centre for Biological Materials. *N. farcinica* was inoculated in brain–heart infusion medium (BHI; Oxoid) at 37 °C. *Mycobacterium smegmatis* mc^2^ 155 was provided by Kanglin Wan (Chinese Center for Disease Control and Prevention, Beijing, China) and grown in Luria–Bertani (LB) medium supplemented with 0.05% Tween 80 (Sigma-Aldrich). *E. coli* DH5α and BL21 (DE3) cells (TransGen Biotech, China) were grown in LB medium at 37 °C. The human epithelial cell line HeLa and Murine MØ cell line RAW 264.7 were grown in Dulbecco’s modified eagle medium (DMEM) containing 10% fetal bovine serum (FBS; all from GIBCO BRL, USA) in a humidified incubator with 5% CO_2_ at 37 °C.

### Plasmids, antibodies, and reagents

The vector pET30a (in our laboratory) was used to express *N. farcinica mce1C* and *mce1D* in *E. coli*. The Mycobacterium shuttle vector pMV261 (provided by Kanglin Wan from the Chinese Center for Disease Control and Prevention) was used to express *N. farcinica mce1C* and *mce1D* in *M. smegmatis*. For expression in mammalian cells, *N. farcinica mce1C* and *mce1D* were cloned into pcDNA6A (provided by Cuihua Liu from the Institute of Microbiology Chinese Academy of Sciences, Beijing, China). The following antibodies were used in this study: anti-p-ERK 1/2, anti-p-JNK, anti-p-P38, anti-p-P65 and anti-p-AKT, MAPK Family Antibody Sampler Kit, anti-P65, anti-AKT (Cell Signaling) and anti-β-actin (TransGen Biotech, China), in addition to the Mouse TNF ELISA Kit (Angle Gen, China), Mouse IL-6 ELISA Kit (Angle Gen, China), and Lipofectamine 3000 (Invitrogen, Carlsbad, CA).

### Plasmid construction

*mce1C* and *mce1E* were amplified from *N. farcinica* IFM10152 genomic and subcloned into prokaryotic expression vector pET30a or pMV261 and eukaryotic expression vector pcDNA6A, respectively. Primers used in amplification are shown in Table 1.

**Table 1:** Primers used for PCR amplification of mce1C or mce1D.

Gene	Primer	Sequence (5′ to 3′)	Vector
*mce1C*	CF	GTAT**CATATG**ATGAACGAGCAGCGATCTCC	*pET30a*
	CR	GCTC**AAGCTT**TCGGGGCGGCTCCTGCACGG	
*mce1D*	DF	GTAT**CATATG**ATGAACAGCTCGAAGATCTT	
	DR	CTAA**CTCGAG**TCGTCCACCTCCCGGAATCG	
*mce1C*	CF	TCAG**GAATTC**ATGAACGAGCAGCGATCT	*pMV261*
	CR	GATC**AAGCTT**TCATCGGGGCGGCTCCTGC	
*mce1D*	DF	TCAG**GAATTC**ATGAACAGCTCGAAGATC	
	DR	GATC**AAGCTT**TCATCGTCCACCTCCCGG	
*mce1C*	CF	GCTC**AAGCTT**ATGAACGAGCAGCGATCTCC	*pcDNA6A*
	CR	GTAT**GAATTC**TCGGGGCGGCTCCTGCACGG	
*mce1D*	DF	GCTC**AAGCTT**ATGAACAGCTCGAAGATCTT	
	DR	GTAT**GAATTC**TCGTCCACCTCCCGGAATCG	

Restriction sites in primers are indicated in bold letters.

### Cloning and expression of recombinant Mce1C and Mce1D

The PCR amplification product were cloned in pET30a to generate pET30a-mce1C or pET30a-mce1D with hexa-histidine tags after being transformed into *E. coli* DH5α. Subsequently, *E. coli* DH5α was grown in LB agar in the presence of kanamycin (50 μg/ml) to enable the recognition of positive clones. The recombinant plasmids were sequenced and then transformed into *E. coli* BL21 cells.

According to the methods^18^, the *E. coli* cells were induced with 0.2 mM isopropyl β-d-1-thiogalactopyranoside (IPTG) at 30 °C for 4 h. The bacteria were sonicated in an ice bath, then centrifuged 12,000 rpm at 4 °C for 20 min. The precipitation and supernatant were collected carefully, and then the expression forms were determined.

### Purification and renaturation of the recombinant Mce1D

N-terminal His-tagged recombinant proteins were purified with a His·Bind Purification Kit (Novagen) according to the manufacturer’s instructions. The purified inclusion bodies were dialyzed in a urea concentration gradient (6, 4, 2, and 0 mol/l) and renatured at 4 °C for 24 h. Renatured protein was transferred to PBS and stored at 4 °C overnight.

### Localization of the Mce1C and Mce1D protein

The locations of the Mce1C and Mce1D were analyzed by using a method previously described^27,28^. Briefly, the cells were cultured in BHI for 16 to 18 h, and lysed via sonication, and cell debris and nonlysed cells were removed through centrifugation at 5,000*g* for 45 min at 4 °C. The supernatant was subjected to ultracentrifugation at 27,000*g* for 1 h at 4 °C. The pellet from this centrifugation step was considered the cell wall fraction, and the supernatant was considered the combined cell membrane and cytosol fractions. Equal amounts of protein (8 µg) from each fraction were subjected to Western blotting using mouse antibodies against Mce1C and Mce1D for analyses of protein expression. The antibodies against Mce1C and Mce1D were prepared as the following: Proteins were separated via 12% SDS-PAGE. After electrophoresis^29^, target proteins were isolated by cutting the gel slices, which were stained by 0.25 mol/l KCl and then grinded. Mice were immunized subcutaneously with the gel three times every 2 weeks, and serum was collected 7 days after the last immunization. Titers of serum were determined by ELISA.

### Immunogenicity of Mce1C and Mce1D proteins

Western blot was carried out with our earlier protocol with slight modifications^18^. The recombinant *E. coli* expressing Mce1C and Mce1D proteins were induced with 0.2 mmol/l IPTG. After sonication and centrifugation, the precipitation was transferred onto polyvinylidene fluoride membranes. Mice and rabbits were immunized subcutaneously with *N. farcinica*, and the antisera were used as primary antibodies and anti-rabbit IgG (Beyotime Biotechnology, China), or anti-mouse IgG antibodies (SouthernBiotech, USA) were used as secondary antibodies to detect recombinant Mce1C and Mce1D proteins.

### Invasion assays

Five-microliter samples of stock suspensions of latex beads (4% w/v, 0.3-μm diameter; Thermo Fisher) were combined with 1 ml of PBS containing 60 μg of mce1D protein; latex beads without protein coating served as controls. The mixtures were incubated for 2 h at 37 °C. HeLa cells were seeded in a 24-well polystyrene tissue culture plate to continue incubation until a cell monolayer formed.

Due to the difficulty of Mce1C protein purification, we used the recombinant bacteria to carry out the experiment. The recombinant *E. coli* expressing Mce1C proteins was cultured in LB medium and then induced with 0.2 mmol/l IPTG at 30 °C for 2 h. The induced *E. coli* was pelleted and resuspended in medium to prepare the inoculum. Recombinant *E. coli* cells were added to the monolayer at a multiplicity of infection (MOI) of 10:1 and incubated at 37 °C for 4 h, washed three times with PBS, and processed for electron microscopy as described previously^30^.

For Mce1D, a 200-μl aliquot of the latex beads coated with mce1D protein or latex beads alone was added to HeLa cells monolayers grown in a 24-well plate. The cells were incubated at 37 °C for 24 h and washed three times with PBS and then processed for electron microscopy.

The cells infected with recombinant *E. coli* or incubated with mce1D protein were fixed with 2% glutaraldehyde, postfixed in 1% osmium tetroxide, and dehydrated with an increasing concentration-graded series of ethanol solutions. Ultrathin sections of the cell samples were cut and then examined by transmission electron microscopy (TEM, HT7700, Japan)^18^.

### Blocking assay

To examine the potentially neutralizing activities of anti-Mce1C and anti-Mce1D antibodies, the ability to inhibit bacterial invasion of epithelial cells was analyzed.

The logarithmic phase *N. farcinica* were incubated with anti-Mce1C, anti-Mce1D, or control serum for 1 h at room temperature. The HeLa cells was cultured and maintained in DMEM with 10% fetal bovine serum. For the assay, HeLa cells were seeded onto 24-well plates for 24 h before use. The assay was performed in triplicate. The cells were infected at an MOI of 10 with *N. farcinica* preincubated with antisera for 1 h at 37 °C. Extracellular bacteria was killed by amikacin (200 μg/ml). Then the HeLa cells were incubated with 1 ml of sterile water for 20 min at 37 °C to release the intracellular bacteria. Serial tenfold dilutions of the lysates were plated on BHI agar for determination of CFU numbers.

### *N. farcinica* infection assay

The infection of HeLa cells was carried out following a protocol with slight modifications^31^. Cells was maintained in DMEM with 10% FCS. Two days before infection, an estimated 2 × 10^5^ cells/well were incubated in a 6-well plate. Overnight cultures of *N. farcinica* was diluted 1:100 in fresh BHI medium and grown to an OD_600_ of 0.8 and then resuspended in DMEM supplemented with 2% FCS. The bacterial suspension directly into each well at an MOI of 10.

### RNA extraction

Total RNA was isolated at 1, 3, and 6 h from *N. farcinica* (IFM10152)-infected HeLa cells and at 2 h for clinical isolates. According to the method^32^, three technical replicates (individual wells) were pooled into one biological replicate. Two biological replicates were used for each time point, and three independent experiments were performed. Before RNA extraction, the cell mixture was sonicated on ice for 2 min. Four treatments of 30 s each were performed to disrupt the cells, and the tubes were placed on ice for 30 s between each treatment. After disruption of the cell mixture, the RNeasy Mini kit (Qiagen) was used to extract total RNA according to the manufacturer’s recommended protocol.

### Real-time PCR

Real-time PCR was performed to analyze the relative levels of the mRNA transcripts, and *secA* was used as an internal control. cDNA syntheses were performed at 37 °C for 15 min and at 85 °C for 5 min using Prime Script reverse transcription reagents (TaKaRa, Japan). Real-time PCR was carried out by using the SYBR Premix Ex Taq II reagents (TaKaRa) and according to the manufacturer’s instructions. The primers for targeted genes are listed in Table 2.

**Table 2:** Genes and primers used in RT-PCR.

Gene	Primer	Sequence (5′ to 3′)	Size (bp)
*mce1C*	Forward	ATGAACGAGCAGCGATCTCCC	311
	Reverse	CCGAGCACGGTGTTGGTCTTG	
*mce1D*	Forward	GGGTGAAGGTGACGATGACGG	82
	Reverse	ACCAGCGAGGGCGAGACGAT	
*secA*	Forward	CCCAGTCCTCCACGTAGCCT	142
	Reverse	CAGCAGCGCACCGTCATCT	

### Construction recombinant *M. smegmatis*

*M. smegmatis* strain mc^2^ 155 was grown in LB supplied with 0.05% Tween 80 until mid-log phase. Cells were then harvested and washed three times with cold 10% glycerol. Next, 50 μl of the competent cells was mixed with 2 µg of pMV261-*mce1C*, pMV261-*mce1D*, or pMV261 vector and then electroporated following the standard setting of 2.5 kV, 1,000 Ω, and 25 μF using the Gene Pulser Xcell apparatus (BioRad). Cells were harvested with 1 ml of BHI and incubated for 2 h at 37 °C with gently shaking, and the culture were plated in BHI plates containing 50 μg/ml kanamycin. The expression of mce1C and mce1D in recombinant *M. smegmatis* was confirmed by using corresponding specific primers and Western blotting.

### Recombinant *M. smegmatis* infection of RAW264.7 cells

RAW264.7 cells were seeded at a density of 2 × 10^5^ cells/well in 24-well plates and cultured for 16 h before infection. The cells resuspended in DMEM supplemented with 2% FCS were infected with mid-log phase recombinant *M. smegmatis* for 2 h at an MOI of 5. After incubation for 2 h, the cells were washed three times with sterile PBS to remove extracellular bacteria, followed by culturing in fresh medium containing 25 μg/ml gentamicin. At various time points, cell culture supernatant was obtained for ELISA. The production of TNF and IL-6 were quantified by the use of ELISA kits. At 24 h postinfection, cells were washed three times with PBS and solubilized with 1 ml of sterile water for 20 min at 37 °C. Then, intracellular survival was determined by plating serially diluted cultures on BHI plates.

### Cell transfection

RAW264.7 cells were seeded at a density of 6 × 10^5^ cells/well in 6-well plates and cultured for 24 h before transfection. Transient transfection was performed with.

Lipofectamine 3,000 according to the manufacturer’s instructions. After 36 h of transfection, the cells were stimulated at an MOI of 10 with *N. farcinica* that *was* inactivated with 0.1% formaldehyde for 24 h. The expressions of Mce1C and Mce1D in RAW264.7 cells after transfection were verified by Western blotting.

### Western blot analysis

After various treatments, RAW264.7 cells were lysed with RIPA buffer supplemented with phosphatase and protease inhibitor (CWBIO, China). Protein were separated by SDS-PAGE and then transferred to polyvinylidene fluoride membranes (Millipore). The membranes were incubated overnight at 4 °C with primary antibodies NF-κB p-P65, P65, p-P38 MAPK, P38, p-ERK1/2, ERK1/2, p-JNK, JNK, p-AKT, AKT, and β-actin. Subsequently, membranes were incubated with HRP-conjugated anti-rabbit IgG (Beyotime Biotechnology, China) or anti-mouse IgG antibodies (Southern Biotech, USA) and developed using the Western Lightning Plus ECL kit (PerkinElmer, USA).

### Statistical analysis

Analyses were performed using the SPSS 22.0. Group means and standard deviations (SDs) were compared with the use of two-sided Student’s *t* tests. *P* < 0.05 was considered to indicate statistical significance.

## Results

### Expression and purification of recombinant protein

**Figure 1:**
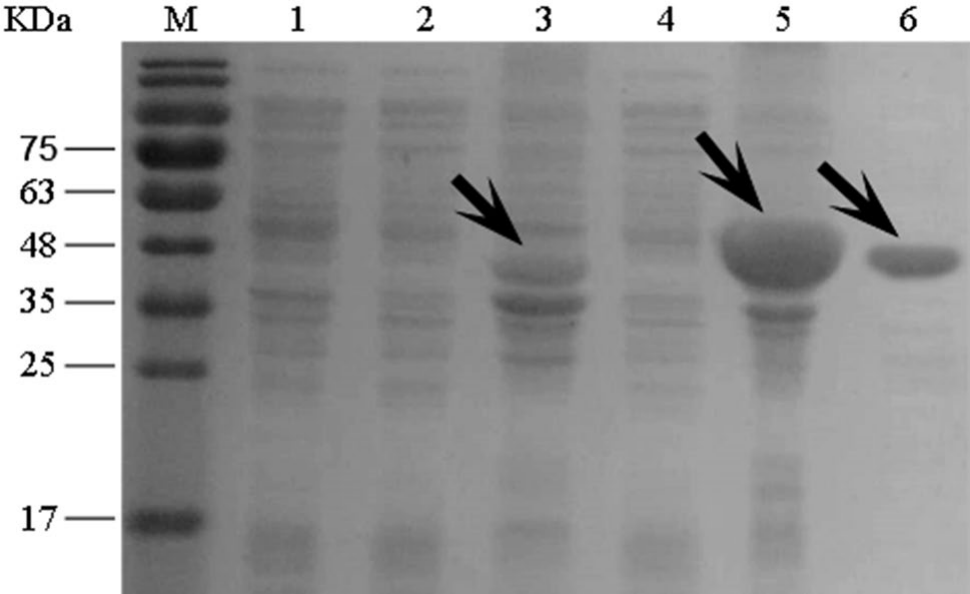
SDS-PAGE analysis of proteins expressed in transformed *E. coli*. **Lane M** Protein marker. **Lane 1** Vector control expression in BL21 cultured for 4 h with IPTG. **Lane 2** and **3** Supernatant and precipitation of recombinant *E. coli* expressing Mce1C proteins cultured for 4 h with 0.2 mmol/l IPTG, respectively. **Lane 4** and **5** Supernatant and precipitation of recombinant *E. coli* expressing Mce1D proteins cultured for 4 h with 0.2 mmol/l IPTG, respectively. **Lane 6** purified Mce1D protein.

Double-enzyme digestion and DNA sequencing confirmed that the pET30a-mce1C and pET30a-mce1D recombinant expression vectors were constructed successfully. Following transformation of *E. coli* BL21(DE3) with pET30a-mce1C and pET30a-mce1D, protein expression induced with 0.2 mmol/l IPTG (30 °C for 4 h) was analyzed by SDS-PAGE. Proteins were detected in the precipitation (Fig. 1). The purified Mce1D proteins were renatured by use of the dialysis method and identified by SDS-PAGE. Purified Mce1D proteins presented as a single band.

### Antigenicity of Mce1C and Mce1D proteins

**Figure 2:**
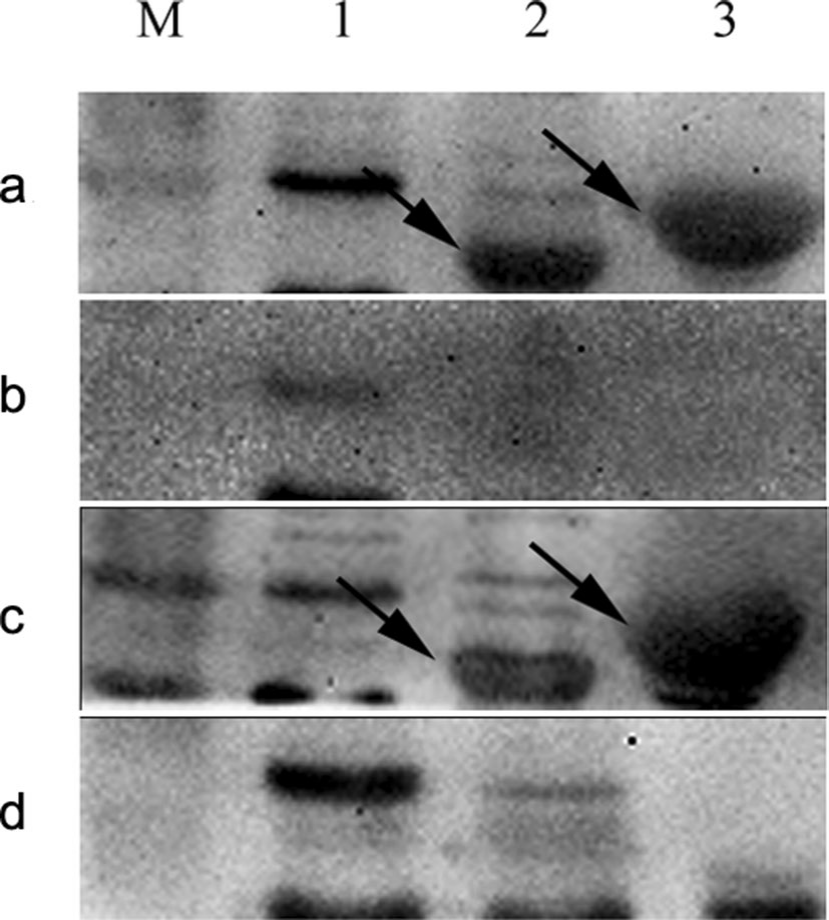
Mce1C and Mce1D protein can be recognized by antisera from mice and rabbits infected with *N. farcinica*. The antigenicity of Mce1C and Mce1D protein was analyzed using Western blotting. **Lane M** Protein marker. **Lane 1** Protein from vector control *E. coli* induced for 4 h with 0.2 mmol/l IPTG. **Lane 2** Protein from recombinant *E. coli* expressing Mce1C protein induced for 4 h with 0.2 mmol/l IPTG. **Lane 3** Protein from recombinant *E. coli* expressing Mce1D protein induced for 4 h with 0.2 mmol/l IPTG. (**a**) Antisera from BALB/c mice infected with *N. farcinica*; (**b**) Antisera from BALB/c mice infected with normal saline; (**c**) Antisera from New Zealand White rabbits infected with *N. farcinica*; (**d**) Antisera from rabbits infected with normal saline.

A number of Mce proteins in *M. tuberculosis* have been found to have immunogenicity and be expressed and elicit antibody responses during infection^17^. Besides, we have found Mce1E protein could be identified by antisera^18^. We hypothesized that Mce1C or Mce1D protein from *N. farcinica* may have antigenicity. The anti-*N. farcinica* sera from human are not available in our laboratory; to make the results more credible, we prepared antisera from different species. As shown in Fig. 2, the immunopositive band was observed when the precipitation of recombinant BL21 expressing Mce1C and Mce1D proteins was immunoblotted with sera from *N. farcinica*-infected mice and rabbits. The recombinant Mce1C and Mce1D proteins can be identified by mouse and rabbit antisera but not by control sera, indicating that Mce1C and Mce1D proteins have immunogenicity and can be expressed during infection.

### Subcellular localization of Mce1C and Mce1D proteins

**Figure 3:**
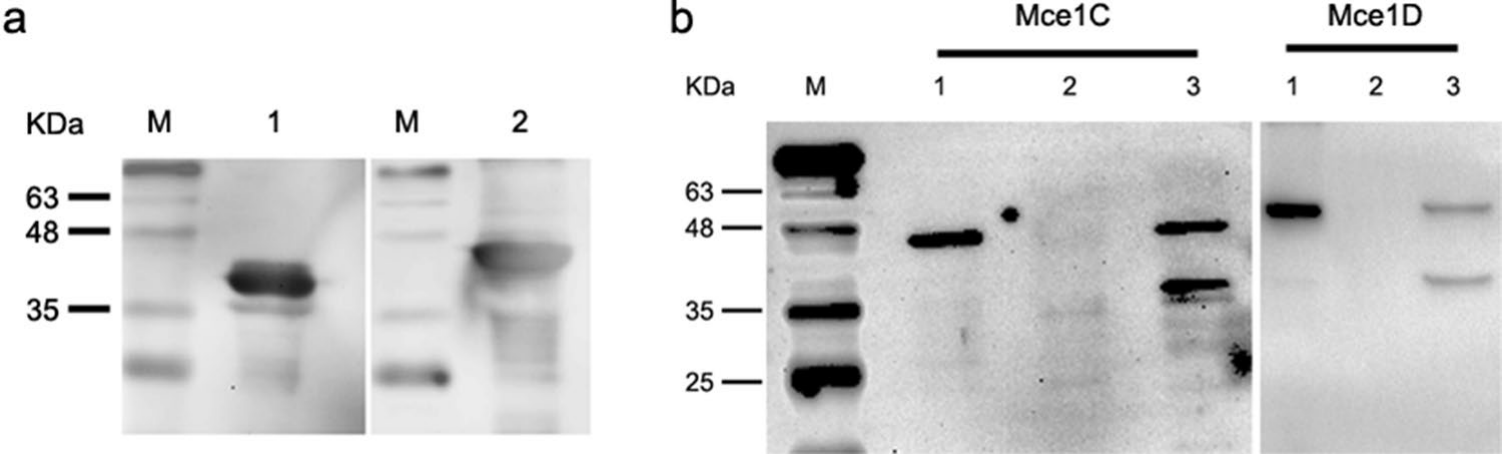
Mce1C and Mce1D were localized in the cell wall. (**a**) The recombinant Mce1C or Mce1D protein from *E. coli* can be identified by anti-Mce1C or Mce1D sera, respectively. **Lane 1** Mce1C protein. **Lane 2** Mce1D protein. (**b**) Subcellular localization of Mce1C and Mce1D protein were analyzed using Western blotting with anti-Mce1C or Mce1D sera at a dilution of 1:4,000. **Lane M** Protein marker. **Lane 1** whole cell lysate. **Lane 2** cytosol and cell membrane. **Lane 3** cell walls.

We hypothesized that Mce1C or Mce1D protein could be localized in the cell wall, which would provide an explanation for the ability to facilitate *N. farcinica* interaction with and invasion of mammalian cells. Since the recombinant Mce1C and Mce1D proteins can be identified by antisera, we believe that these two proteins must be expressed in *N. farcinica*. To test our hypothesis, first anti-Mce1C or Mce1D protein sera were generated (Fig. 3a) and then *N. farcinica* was subjected to cell fractionation experiments. The Mce1C and Mce1D protein was detected in the cell wall fraction (Fig. 3b), indicating that Mce1C or Mce1D is associated with the cell wall fraction.

### Invasion of HeLa cells by recombinant *E. coli* expressing Mce1C protein

**Figure 4:**
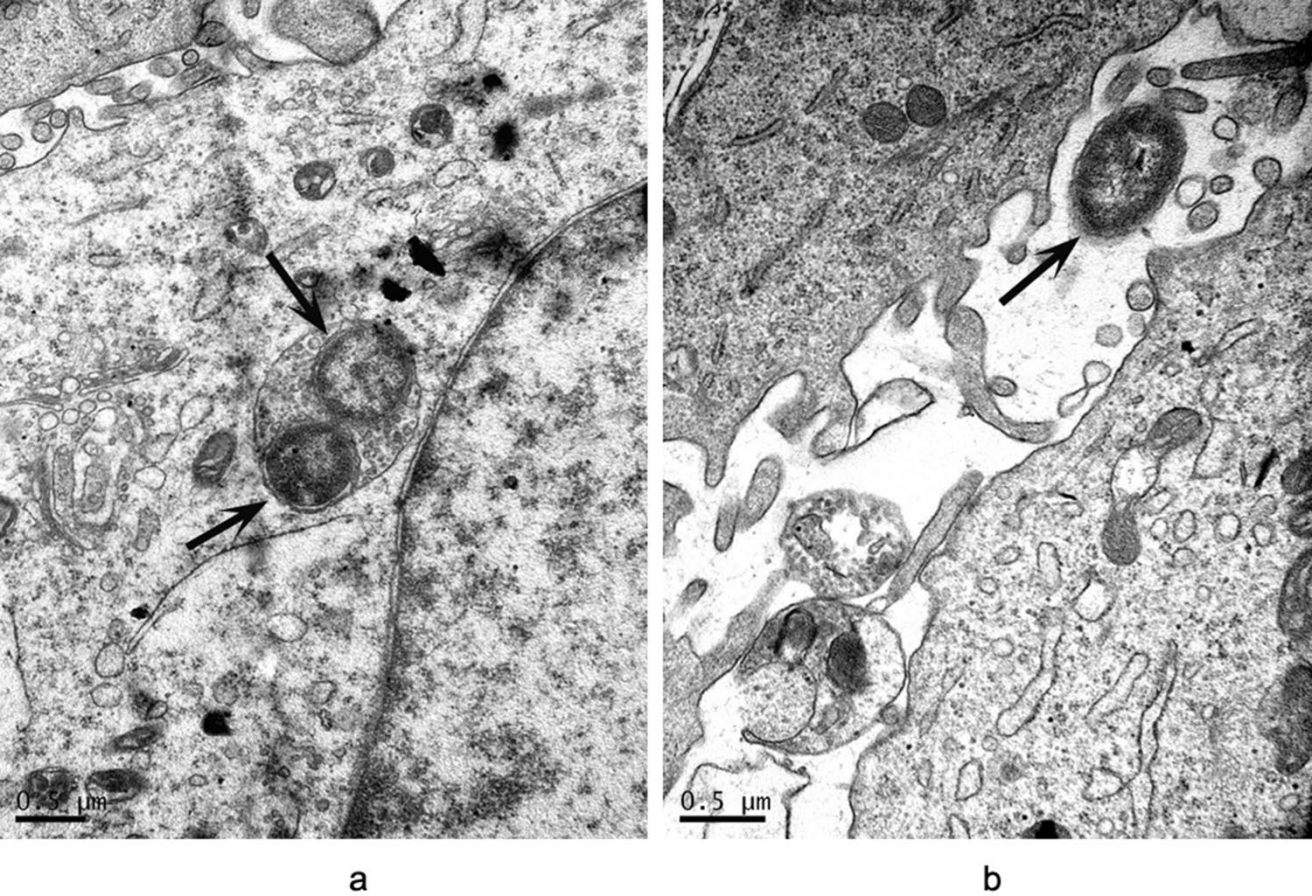
Invasion of HeLa cells by recombinant *E. coli* expressing Mce1C. (**a**) HeLa cells were infected with induced *E. coli* expressing Mce1C for 4 h and then analyzed by electron microscopy. (**b**) Negative control (*E. coli*-pet30a). Recombinant *E. coli.* were marked by arrows. Bars = 0.5 μm.

Transmission electron microscopy (TEM) confirmed the invasion of HeLa cells by recombinant *E. coli* expressing Mce1C protein. The results show that induced *E. coli* expressing Mce1C protein was able to enter the HeLa cells (Fig. 4a) and internalized recombinant *E. coli* was observed within the cytoplasmic vacuoles. The *E. coli* (pET30a) used as a negative control failed to enter the HeLa cells (Fig. 4b).

### Invasion of HeLa cells by Mce1D-coated latex beads

**Figure 5:**
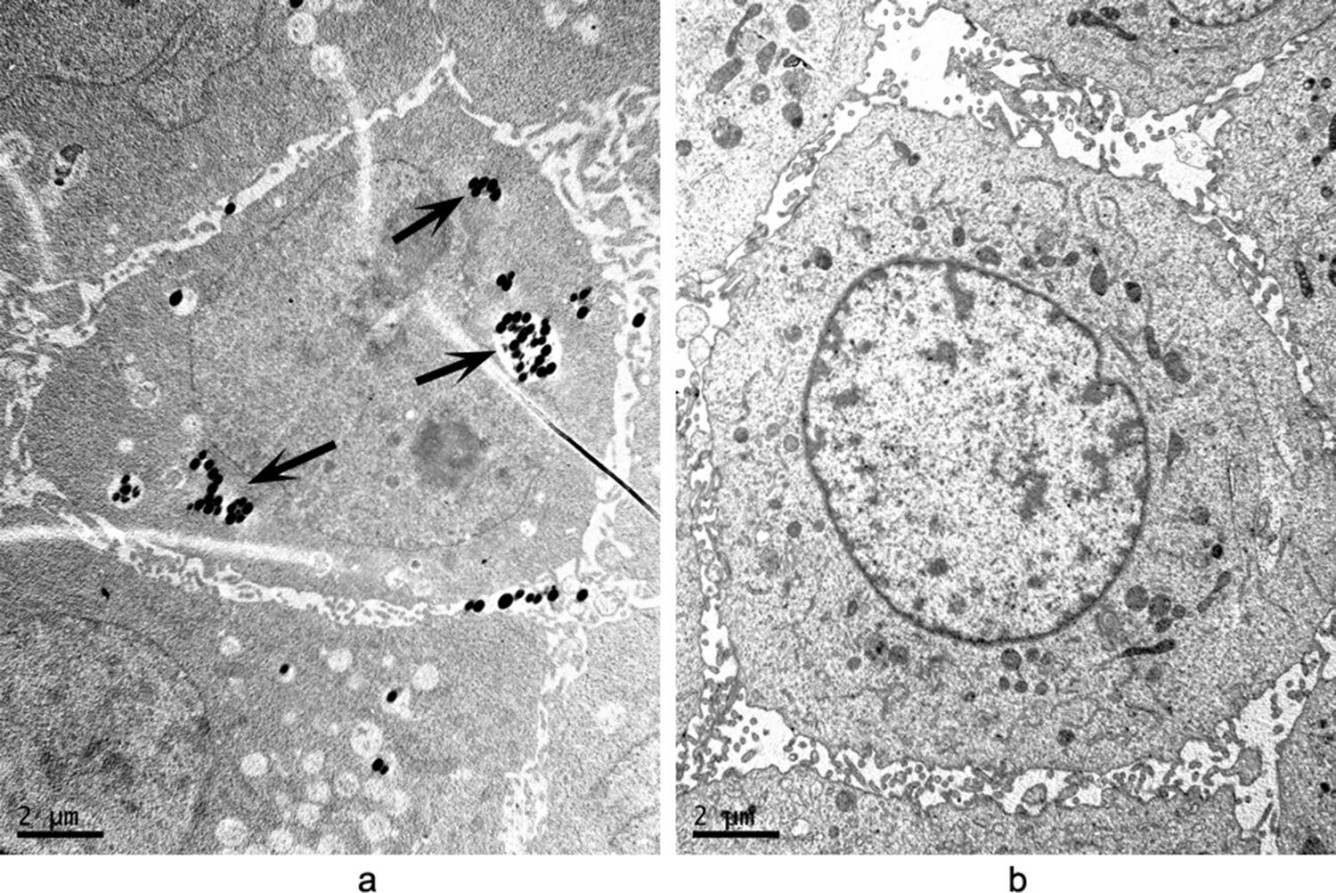
Latex beads coated with Mce1D was internalized by HeLa cells. (**a**) Latex beads coated with mce1D were internalized completely by HeLa cells. (**b**) Latex beads without protein coating were not observed in HeLa cells. Both solitary beads (arrow) and clusters of beads were present in HeLa cells (some marked by arrows). Bars = 2 μm.

TEM has been used previously to demonstrate that latex beads coated with Mce1E are internalized by HeLa cells^18^. TEM examination results confirmed that Mce1D-coated beads invaded the HeLa cells. After 24 h of incubation, the Mce1D-coated beads were observed within vacuolar compartments, whereas uncoated latex beads were not (Fig. 5).

### Effects of anti-Mce1C or -Mce1D sera on the ability of *N. farcinic*a to invade HeLa cells

**Figure 6:**
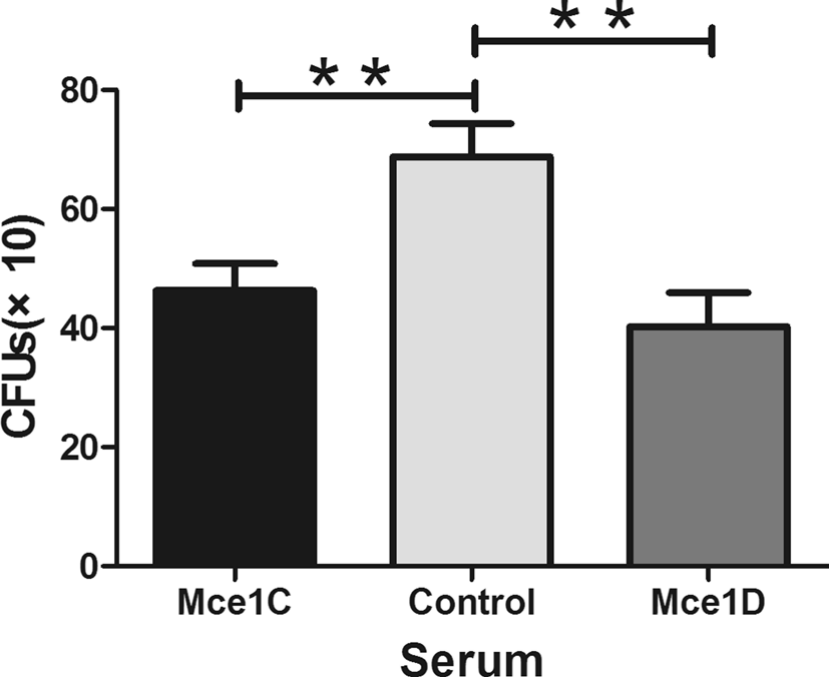
Inhibitory effects of anti-Mce1C or Mce1D sera on *N. farcinica* invasion of HeLa cells. The HeLa cells were infected with *N. farcinica* preincubated with anti-Mce1C or Mce1D sera or control sera at a dilution of 1:200 in DMEM. The HeLa cells were washed three times with PBS 1 h postinfection and then treated with Amikacin (200 μg/ml) for 1.5 h to kill extracellular bacteria. Lysates were plated on BHI plates to determination the viable intracellular bacteria. The assays were performed in triplicate and the results are expressed as the means ± SD. **P < .01.

We found that Mce1C and Mce1D protein could be expressed and elicit antibodies; therefore, we investigated whether the antisera could inhibit the invasion of *N. farcinica* into HeLa cells. As shown in Fig. 6, after incubation with antisera, there are less CFUs in the HeLa cells, indicating that the invasion of *N. farcinica* was inhibited partially by the antisera of Mce1C and Mce1D proteins.

### Analysis of mce1C and mce1D mRNA level during early infection of HeLa cells

**Figure 7:**
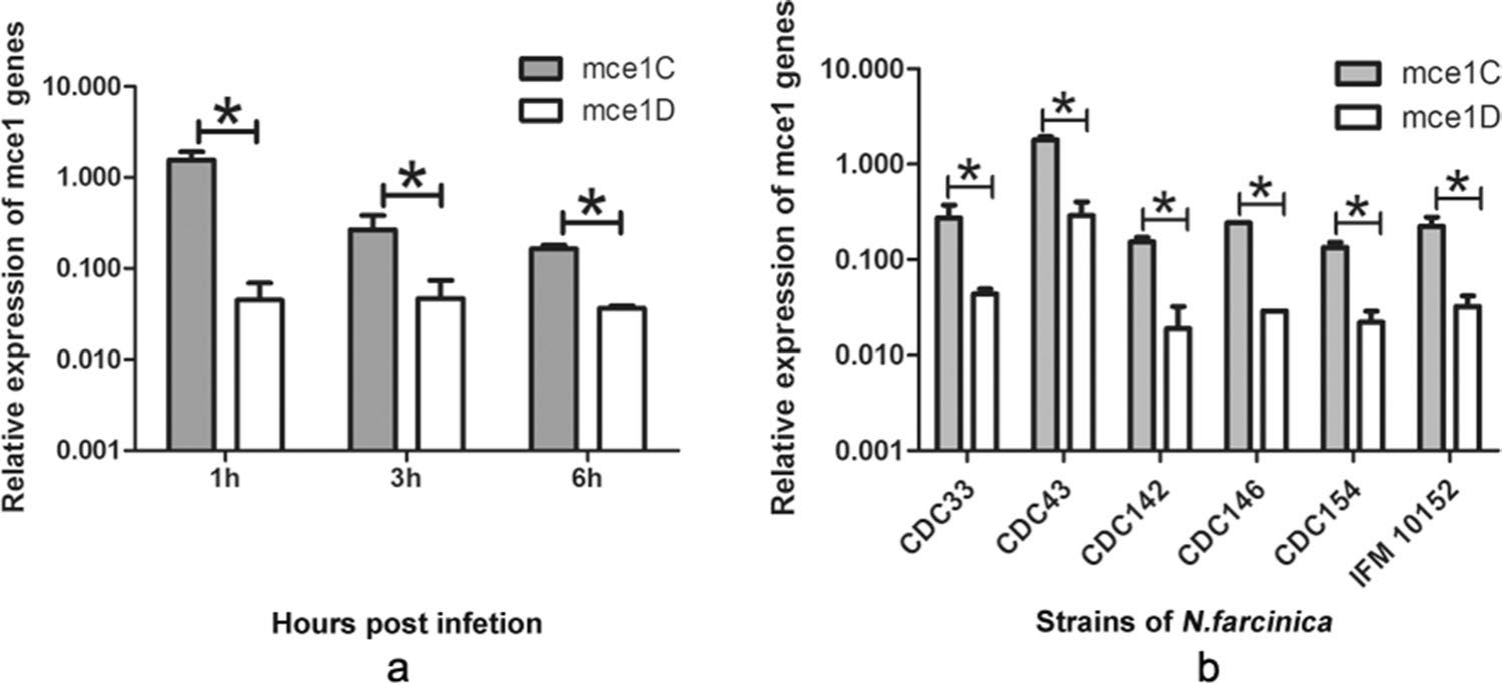
Gene expression level of mce1C and mce1D during infection. (**a**) HeLa cells were infected with the standard strain IFM10152 and the mRNA level of mce1C and mce1D at 1, 3, and 6 h postinfection was determined using RT-PCR. (**b**) HeLa cells were infected with five clinical isolates of *N. farcinica*, and the expression level of mce1C and mce1D were measured at 2 h postinfection. The assays were performed in triplicate and the results are expressed as the means ± SD. *P < .05.

Mce1C and Mce1D proteins were expressed at the protein level in this study, we further verified the expression at mRNA level during infection. Five clinical isolates of *N. farcinica* and the standard strain *N. farcinica* IFM10152 were used in this study. RT-PCR analysis revealed expression profiles of mce1C and mce1D genes during the infection of HeLa cells. As shown in Fig. 7a, both Mce1C and Mce1D were expressed at the mRNA level, and the expression level of mce1C is higher than that of mce1D during infection. As shown in Fig. 7b, the expression level of mce1C is higher than that of mce1D for all clinical isolates, and the strain CDC43 showed the highest expression of mce1C or Mce1D compared with other strains.

### Mce1C and Mce1D inhibit the production of TNF-α and IL-6 and promote the survival of *N. farcinica* in macrophages

**Figure 8:**
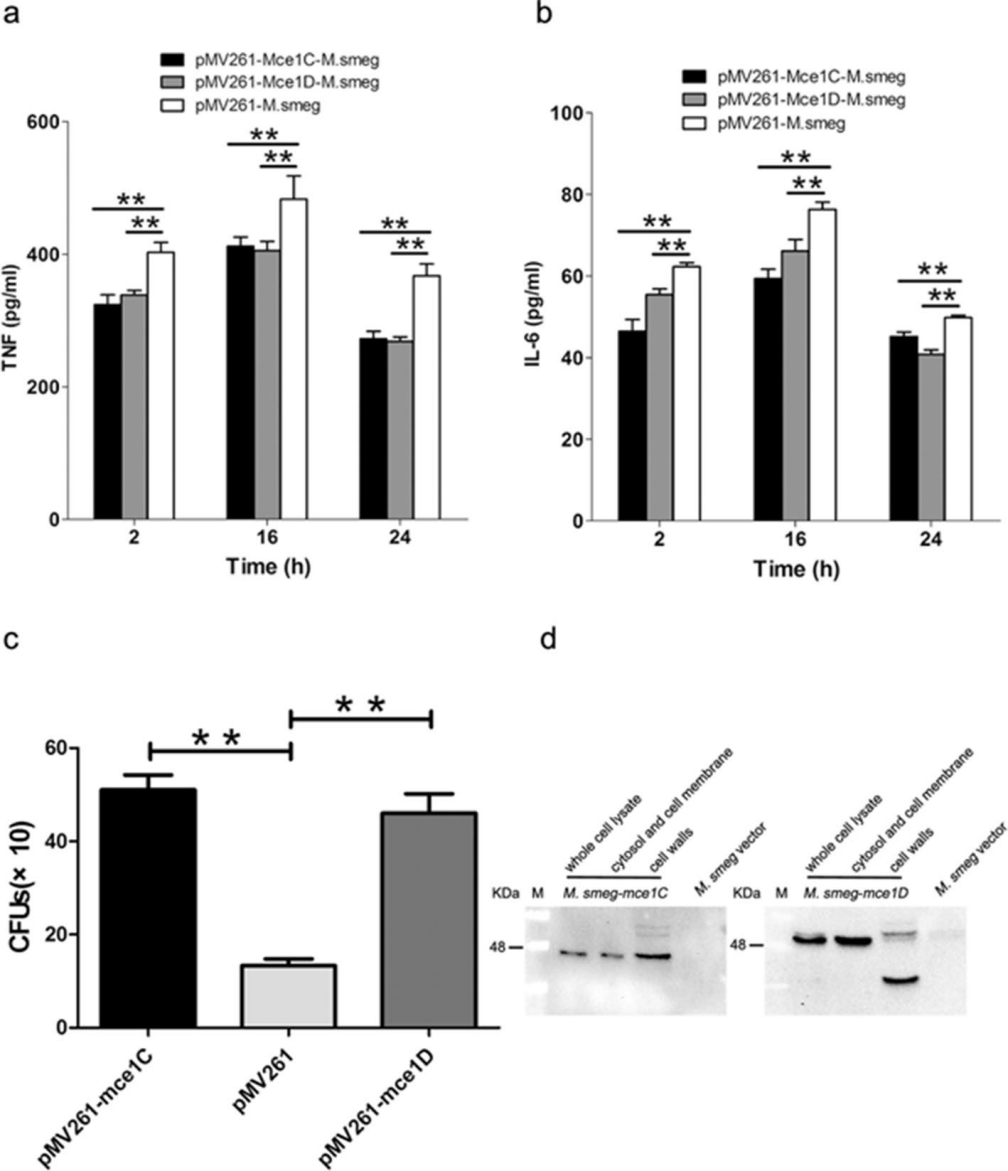
*N. farcinica* Mce1C and Mce1D suppresses production of TNF and IL-6 and promotes the survival of *M. smegmatis* in RAW264.7 macrophages during infection. **a and b** ELISA analysis of TNF-α (**a**) and IL-6 (**b**) protein in culture media from RAW264.7 cells infected with *M. smegmatis* expressing Mce1C, Mce1D, or vector for 2 to 24 h. (**c**) Expression of *N. farcinica* Mce1C or Mce1D in *M. smegmatis* promotes the survival of *M. smegmatis* in RAW264.7 cells. RAW264.7 cells were infected with recombinant *M. smegmatis* or vector control for 24 h, and then the intracellular survival of *M. smegmatis* was determined. (**d**) *N. farcinica* Mce1C or Mce1D was expressed in recombinant *M. smegmatis*. The expression of Mce1C or Mce1D in *M. smegmatis* was analyzed using Western blotting with the anti-Mce1C or Mce1D sera as the primary antibody. Data are representative of three independent experiments (means ± SD). **P < .01.

Bacterial infection first activates the host’s innate immune response, producing a series of inflammatory cytokines, such as TNF-α, IL-6, and IL-10. These cytokines are essential for recruitment and activation of immune cells to respond to infection^20^. Given that *mce1C* and *mce1D* contribute to *N. farcinica* pathogenicity, little is known about the roles of regulating innate immune response, especially the production proinflammatory cytokines. *M. smegmatis* is a kind of fast-growing gram-positive nonpathogenic mycobacterium. Like *N. farcinica*, the strain belongs to actinomycetes and contains mce operon; the strain is widely used to study the protein function of *Mycobacterium*^26,33,34^. Recombinant *M. smegmatis* mce1C or *M. smegmatis* mce1D was constructed successfully, and the expression of Mce1C and Mce1D was verified Western blotting (Fig. 8d). To examine the ability of *N. farcinica* Mce1C and Mce1D to modulate cytokine expression, we analyzed the production of proinflammatory cytokines in macrophages infected with *M. smegmatis* Mce1C or Mce1D by using ELISA. As shown in Fig. 8a,b, expression of Mce1C or Mce1D in *M. smegmatis* attenuated the production of TNF-α and IL-6. To determine whether Mce1C or Mce1D could promote bacterial survival in macrophages, The bacterial loads was assessed in RAW264.7 cells 24 h postinfection. Results showed that the CFUs of bacteria in cells were higher than those of the control group, suggesting that Mce1C and Mce1D played an important role in the survival of *M. smegmatis* in macrophages. These results indicate that Mce1C and Mce1D can suppress the expression of proinflammatory cytokines and promote the survival of *M. smegmatis* in macrophages.

### Mce1C and Mce1D inhibited the NF-κB and MAPK pathways

**Figure 9:**
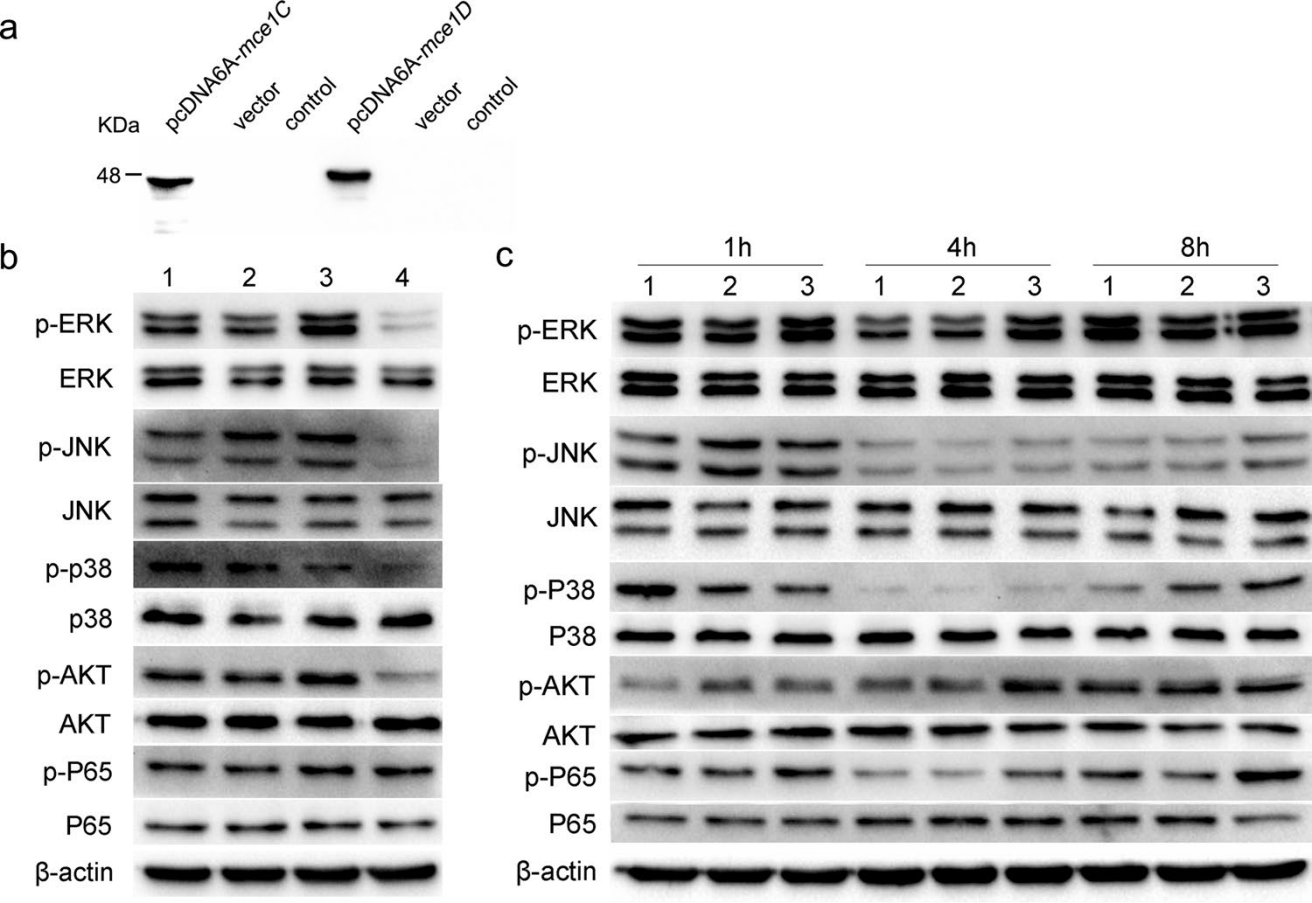
Mce1C and Mce1D inhibite the NF-κB and MAPK signal pathways. (**a**) The expression of *N. farcinica* Mce1C or Mce1D in RAW264.7 cells were determined by western blot. (**b**) RAW264.7 cells were transfected with the Mce1C, Mce1D or vector. The protein levels of p-P65, P65, p-ERK1/2, ERK1/2, p-P38, P38, p-JNK, JNK, p-AKT and AKT were determined by western blot 24 h after transfection. **1**: RAW264.7 cells transfected with pcDNA6A-mce1C; **2**: RAW264.7 cells transfected with pcDNA6A-mce1D; **3**: RAW264.7 cells transfected with pcDNA6A vector; **4**: RAW264.7 cells control. (**c**) (**1**) RAW264.7-mce1C, (**2**) RAW264.7-mce1D, or (**3**) RAW264.7-vector cells were stimulated with *N. farcinica* (MOI = 10) for the indicated time periods (1 h, 4 h, or 8 h) and the phosphorylate of proteins above was analyzed by western blot.

To asses the potential role of Mce1C and Mce1D in modulating host innate immunity, we further investigated the activation of signaling pathways that may contribute to differential cytokine release in RAW264.7 cells after recombinant *M. smegmatis* infection and the activation status of NF-κB and MAPK pathways were determined by Western blotting. First, RAW264.7 cells were transfected with mce1C, mce1D, or pMV261 vector. Western blotting confirmed the expression of Mce1C or Mce1D protein in RAW264.7 cells (Fig. 9a). The results indicated that transient expression of *N. farcinica* mce1C in RAW264.7 cells inhibited ERK1/2, JNK, AKT, and P65 phosphorylation and transient expression of *N. farcinica* mce1D in RAW264.7 cells mainly inhibited ERK1/2, AKT, and P65 phosphorylation (Fig. 9b). Interestingly, the phosphorylation of P38 was induced by Mce1C and Mce1D. To further examine whether Mce1C or Mce1D suppressed signaling pathway in macrophages after *N. farcinica* induction, the RAW264.7 cells were stimulated with *N. farcinica* after transfection of *N. farcinica* mce1C, mce1D, or pMV261 vector in cells. The results showed that the phosphorylation of ERK1/2, JNK, AKT, and P65 was inhibited to varying degrees in RAW264.7 cells for the indicated time periods (Fig. 9c), which further strengthens the preceding results. Interestingly, the phosphorylation of P38 was activated by Mce1C mainly at an early induction time, which consisted of the preceding assays, and the phosphorylation of P38 was obviously inhibited at 8 h after induction. These results indicate that both Mce1C and Mce1D inhibit the activation of AKT/NF-κB and MAPK signaling pathways and suppress the innate immune response. In addition, the roles of Mce1C and Mce1D in modulating P38 and JNK signaling pathways differed.

## Discussion

Invading host cells is the initial step in the pathogenesis for intracellular pathogens^30^ and survival in macrophages is also essential to the pathogenesis of *N. farcinica*. To facilitate the invasion function, mce proteins may be located on the cell surface, suggesting that these proteins play an important role in the host–pathogen interactions. In this study, we mainly studied the interaction between Mce1C or Mce1D protein and host cells and gave a comprehensive account of the role of Mce1C or Mce1D proteins in pathogenicity.

Recent reports describing the role of Mce proteins in *Leptospira* invasion of host tissues indicate that Mce proteins in *Leptospira* may support *Leptospira* adhesion and invasion in a manner similar to that seen for *M. tuberculosis*^35^. Previously, our study showed that latex beads coated with *N. farcinica* Mce1E protein entered HeLa cells.

In this study, Mce1C and Mce1D proteins were cloned and expressed and antisera were prepared. Western blot analyses demonstrated that Mce1C and Mce1D protein was observed in the cell wall fraction with antisera and was undetected in other fraction which is consistent with our hypothesis. It appears there are two bands in cell wall samples in Fig. 3b. Mce1C or Mce1D may exists in different forms in the cell wall or these two proteins broken during the process of processing the cell wall. Next, we used antisera from two different species to verify the antigenicity of this protein. The Mce1C and Mce1D proteins reacted with antibodies in antisera from mice and rabbits, showing that Mce1C and Mce1D are expressed during the course of infection and are immunogenic, eliciting antibody responses in the host, which means that these two proteins must have participated in the infection process. In the present study, we further studied the entry of *N. farcinica* Mce1C and Mce1D, encoded by *mce1C* and *mce1D* of the *mce1* operon, into mammalian cells and the mRNA transcription during infection of HeLa cells. Since HeLa cells are not phagocytes, the entry of the latex beads or recombinant *E. coli* into the cells can be attributed to the function of the protein. Consistent with earlier studies in *M. tuberculosis*^30^, recombinant *E. coli* expressing Mce1C protein of *N. farcinica* can enter the HeLa cells and the internalized recombinant *E. coli* was observed within vacuolar compartments. Also, the present results show that *N. farcinica* Mce1D induced the entry and internalization of associated latex beads by HeLa cells within vacuolar compartments, which is similar to other reports^18,36,37^. These results indicate that Mce1C and Mce1D facilitate the interaction and internalization of *N. farcinica* by mammalian cells. The peptides in Mce1C and Mce1D proteins, which play key roles in invasion function, need to be identified in the next study. At the level of mRNA transcription of mce1C and mce1D, our results show that mce1C and mce1D were expressed at 1, 3, and 6 h postinfection of HeLa cells in different clinical strains which further strengthens the results showing that Mce1C and Mce1D were expressed during the infection course. Results also showed that the expression level of mce1C is higher than that of mce1D. In view of the fact that Mce1C and Mce1D proteins can stimulate the host to produce antibodies and facilitate the invasion of *N. farcinica* into mammalian cells, we next verified whether the antiserum of the protein can block and neutralize the invasion function. The results demonstrate that anti-Mce1C or -Mce1D antisera inhibited the invasion of *N. farcinica* into HeLa cells than did the control sera. The findings suggest that antibodies to Mce1C or Mce1D play a protection role against *N. farcinica* infection and are of great significance to further screen monoclonal antibodies of anti-Mce1C or -Mce1D with block function. Shin et al.^38^ shown that human anti-HBHA IgM antibodies recognize the methylated lysine motifs of HBHA and effectively inhibit the invasion of bacteria into A549 cells.

In these assays, we mainly focused on the potential role in invasive function of Mce1C and Mce1D on mammalian cells, and we further studied the function of innate immune regulation involved in its interaction with macrophages after the pathogen entered macrophage cells. Macrophages are important innate immune cells that facilitate the clearance of intracellular bacteria. Besides, macrophages could establish various mechanisms to respond to the invasion of pathogens^39^.

In the present work, we found that Mce1C or Mce1D serves as a virulence factor contributing to the survival of recombinant *M. smegmatis* Mce1C or Mce1D in macrophages (Fig. 8c). Macrophages can secrete proinflammatory cytokines, such as TNF and IL-6, which can enhance the innate immune response and activate the corresponding T cells, thereby enhancing the host’s ability to remove infected bacteria^25,40^. *M. tuberculosis* could subvert immune responses and avoid the elimination of the bacilli within macrophages by regulating innate immune signaling pathways^20^. We demonstrated that the *M. smegmatis* expressing Mce1C or Mce1D protein could decreased the expression of pro-inflammatory cytokines, such as TNF-α (Fig. 8a) and IL-6 (Fig. 8b), in RAW264.7 cells, which in turn inhibit the development of efficient immune responses and contribute to the survival of *M. smegmatis*.

There are several reports that pathogens can produce effector molecules to suppresses inflammatory signal pathways. Yersinia effector YopJ targets and downregulates both the NF-κB and MAPK signal pathways^41^. Shigella flexneri effector OspG can suppress the activation NF-κB signal pathway by affecting the degradation phospho-IκBα^42^. Our data revealed that Mce1C and Mce1D can inhibit the activation of both MAPK and NF-κB pathways induced by *N. farcinica* (Fig. 9) in RAW264.7 cells. Previous studies showed that an intricate balance of ERK 1/2 and P38 MAPK pathways determines the levels of proinflammatory and anti-inflammatory cytokines and that activation of ERK1/2 mainly leads to expression of TNF-α by macrophages, whereas IL-10 secretion is generally dependent on P38 MAPK activation^43,44^. Interestingly, we observed that Mce1C induced phosphorylation of P38 MAPK relative to transfection of pMV261 vector in macrophages. We also detected the activation of P38 at 1 h after stimulation, and P38 phosphorylation was inhibited after 8 h of stimulation. As for ERK 1/2, The phosphorylation of ERK 1/2 in RAW264.7 cells expressing Mce1C protein was inhibited at the beginning of stimulation, but there was no significant difference in the phosphorylation level of ERK 1/2 relative to RAW264.7 cells transfected with empty vectors at 8 h after stimulation. Our results demonstrate that RAW264.7 cells transfected with Mce1C under the stimulation of *N. farcinica* induced a complex regulatory mechanism between ERK and P38. NF-κB and MAPK signaling pathways are downstream of TLR-2 and TLR-4, and TAK1, downstream of TLR, and affect MAPK and NF-κB signaling pathways by phosphorylating IKK and MAPK, thereby inducing the production of inflammatory factors^39^. Whether *N. farcinica* Mce1C or Mce1D suppresses the activation of NF-κB and MAPK signal pathways is related to TLR-2/TLR-4 is still unknown.

To our knowledge, this is the first report that *N. farcinica* Mce1C and Mce1D proteins suppress the expression of proinflammatory cytokines and inhibit the NF-κB and MAPK signaling pathways, thereby inhibiting the innate immune response. Whether the inhibition of the NF-κB and MAPK signaling pathways is directly related to the decrease of proinflammatory cytokine TNF and IL-6 expression needs to be further verified, and the mechanism by which the protein inhibits NF-κB and MAPK signaling pathways is under investigation.

In conclusion, the present work reveals that Mce1C and Mce1D are *N. farcinica* virulence factors. Our study demonstrates that mce1C and mce1D, encoded by the *mce1* operon of *N. farcinica*, facilitated the entry and internalization of *E. coli* expressing Mce1C protein and latex beads coated with Mce1D protein, respectively, by nonphagocytic mammalian (HeLa) cells, and these two proteins could be expressed and are immunogenic during infection. In addition, Mce1C and Mc1D were detected in the cell wall fraction. Further, *N. farcinica* Mce1C and Mc1D might regulate the NF-κB and MAPK signaling pathways to suppress host innate immune responses and promote intracellular survival of bacteria in the macrophages, providing novel insights into the mechanism of the interaction between *N. farcinica* and host cells and necessary information for the possible prevention of *N. farcinica* infection.

## Supplementary information

Supplementary Information.
