# Supplementary material for: Mce1C and Mce1D facilitate N. farcinica invasion of host cells and suppress immune responses by inhibiting innate signaling pathways
Source: Sci Rep. 2020 Sep 10;10:14908. doi: 10.1038/s41598-020-71860-8 (PMC7484815; doi:10.1038/s41598-020-71860-8)
Supplement: Supplementary file 1 — Supplementary Information. [file 41598_2020_71860_MOESM1_ESM.pdf]

Mce1C and Mce1D Facilitate *N. farcinica* Invasion of Host Cells and Suppress Immune Responses by Inhibiting Innate Signaling Pathways

Xingzhao Ji[1], Xiujuan Zhang[2], Lina Sun[1], Xuexin Hou[1], Jingdong Song[5], Xiaoluo Tan[3], Han Song[1,4], Xiaotong Qiu[1], Minghui Li[1], Lu Tang[6], Lichao Han[1], Zhenjun Li[1*]

[*]Corresponding authors: Zhenjun Li, Mailing address: National Institute for Communicable Disease Control and Prevention, Chinese Center for Disease Control and Prevention, State Key Laboratory of Infectious Disease Prevention and Control, 155 Changbai Road Changping District 102206, Beiing, People's Republic of China. Phone: 8610 58900760. Fax: 8610-58900724. E-mail: lizhenjun@icdc.cn

Figure S1

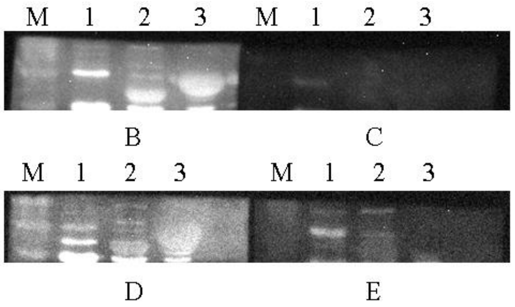

Figure S1 Western blot analysis of recombinant Mce1C and Mce1D proteins. **Lane M**, protein marker. **Lane 1**, the precipitation of vector control BL21 cultured for 3 h with 0.2 mM IPTG. **Lane 2**, precipitation of recombinant BL21expressing Mce1C proteins cultured for 3 h with 0.2 mM IPTG. **Lane 3**, precipitation of recombinant BL21expressing Mce1D proteins cultured for 3 h with 0.2 mM IPTG. **B**: proteins immunoblotted with sera from BALB/c mice infected with N. farcinica; **C**: proteins immunoblotted with sera from BALB/c mice infected with normal saline; **D**: proteins immunoblotted with sera from New Zealand white rabbits infected with N. farcinica; **E**: proteins immunoblotted with sera from New Zealand white rabbits infected with normal saline. Images (B and C) gathered at the same time, from the same locations, and exposure at the same time in the machine, the same as images(D and E).

Figure S2

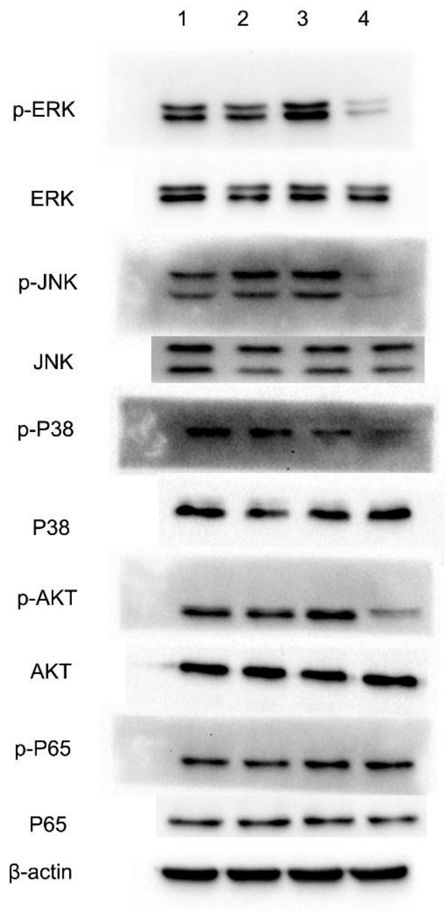

Figure S2 RAW264.7 cells were transfected with the Mce1C, Mce1D or vector. The protein levels of p-P65, P65, p-ERK1/2, ERK1/2, p-P38, P38, p-JNK, JNK, p-AKT and AKT were determined by western blot 24h after transfection. 1: RAW264.7 cells transfected with pcDNA6A-mce1C; 2: RAW264.7 cells transfected with pcDNA6A-mce1D 3: RAW264.7 cells transfected with pcDNA6A vector 4: RAW264.7 cells control.

Figure S3

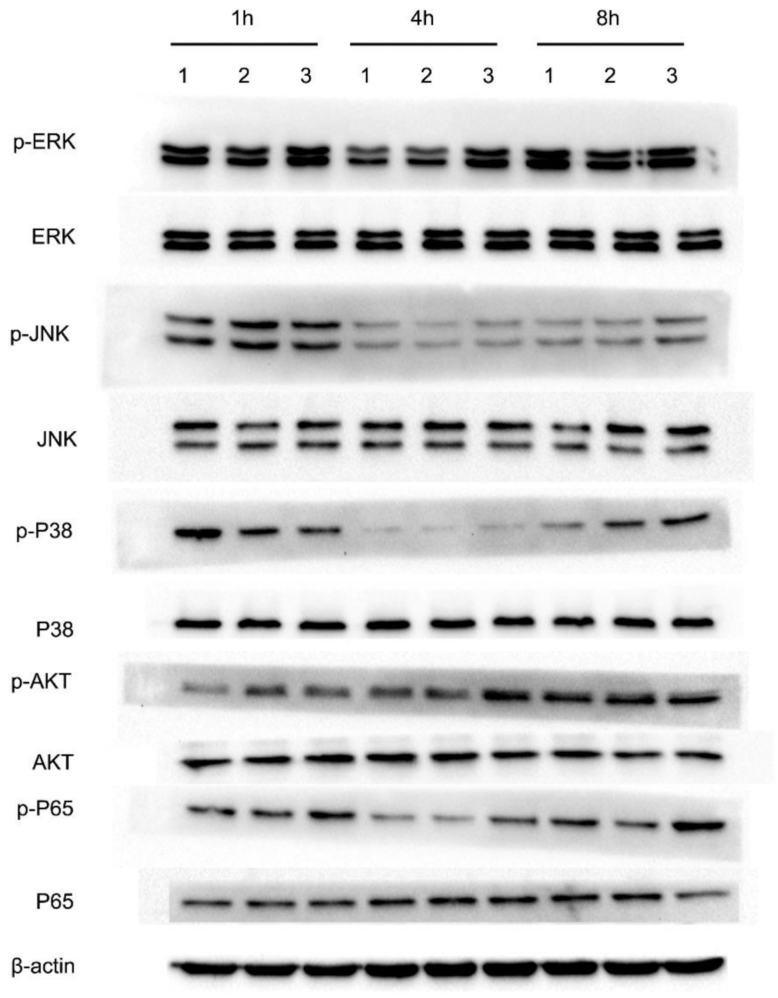

Figure S3 **1** RAW264.7-mce1C, **2** RAW264.7-mce1D, or **3** RAW264.7-vector cells were stimulated with *N.farcinica* (MOI = 10) for the indicated time periods (1h, 4h or 8h) and the phosphorylate of protein above were analyzed by western blot.
